# Comparative Analysis of Lighting Characteristics and Ultraviolet Emissions from Commercial Compact Fluorescent and Incandescent Lamps 

**Published:** 2016-10-23

**Authors:** Mahtab Azizi, Rostam Golmohammadi, Mohsen Aliabadi

**Affiliations:** ^a^ Department of Occupational Health, School of Public Health, Hamadan University of Medical Sciences, Hamadan, Iran; ^b^ Center of Excellence for Occupational Health, Research Center for Health Sciences, Hamadan University of Medical Sciences, Hamadan, Iran

**Keywords:** Compact fluorescent lamp, Ultraviolat emissions, Lighting characteristics, Incandescent lamp

## Abstract

**Background:** Some characteristics of lighting sources such as color properties and ultraviolet emissions have important roles on visual and non-visual health effects of lighting. This study aimed to investigate the light emissions of some compact fluorescent lamps (CFLs) and incandescent lamps commercially available to the Iranian consumers.

**Methods:** Sixty lamps included 48 single envelope CFLs, and 12 incandescent lamps available in the electrical devices markets (in the west of Iran) were randomly selected from famous manufacturers between 2014 and 2015. Lighting characteristics and ultraviolet (UV) emissions were measured using spectroradiometer and calibrated radiometer, respectively. Data analysis was performed using SPSS16 software.

**Results:** Color-rendering indexes of the studied lamps were above 80, which showed good color properties. The daylight CFLs had more desirable and natural color temperature (near to 5000 ^0^k) compared with the other types of the studied lamps. Occupational exposures for periods up 8 h to UVB from the studied lamps at distances up to 0.25 m were more than the recommended limits. Moreover, public exposures for periods up 16 h to UVB from the studied lamps at any distances up to 2 m were more than the recommended limits.

**Conclusions:** Warm white lamps are suitable for homes usage, while daylight lamps can be used for offices rooms. Occupational exposure to single envelope CFLs near the body at distances of less than 25 cm can result in overexposure to actinic UV. Moreover, CFLs must be used at distances greater than 200 cm for public exposure.

## Introduction

In last decades, lighting researches tried to identify better solutions for achieving good artificial lighting^1,2^. In this way, new lighting sources and lighting system designs have been developed^3^. Compact fluorescent lamps (CFLs) as one of the new lighting *technologies* are the group of fluorescent lamps designed to replace with incandescent lamps. The use of CFLs in indoor environments is rising because they present considerable energy and cost savings, smaller sizes, variety of designs and lower costs^4^. However, in terms of lighting quality and visual comfort, these new lighting sources should be investigated^5^. Main characteristics of artificial lighting source including color rendering index(CRI), correlated color temperature (CCT) and spectral power distribution (SPD) can have major contributions on lighting quality and visual comforting indoor environments^6^.

The measurement of light’s ability to render suitably colors is called the color-rendering index (CRI). From 0-100, it describes how a light source makes the color of an object appear to human eyes and how well subtle variations in color shades are revealed. The CRI rating above 80 shows the lighting lamps have good color properties^7^. Moreover, the color appearance of lighting sources is defined in terms of color temperature and is measured in terms of degrees Kelvin. Lighting sources with the higher color temperature appear the cooler while lighting sources with the lower color temperature appear the warmer^7,8^.

Spectral power distribution(SPD) is used for describing visible spectrum of lighting sources. This quantity shows the radiant power emitted by the source at each wavelength over the visible region (380 to 760 nm). Lamp manufacturers present the SPD curves of specific light sources. The incandescent lamp frequently has high power in the longer wavelengths (above 650 nm) of the visible spectrum and therefore, effectively renders red colors. The fluorescent lamp has high power in the short wavelength of the visible spectrum (below 450 nm) and therefore, mainly renders blue colors^9^.

*In terms of CRI,* compact fluorescent lamps* are classified in 1A and 1B groups and incandescent lamps are classified in 1A group*^10^*. The color rendering indexes of incandescent lamps are from 99.2% to 99.9%*^11^. CRI values in cool white lamps are from 82% to 98.8% ^12^. *CRI values in CFLs and incandescent lamps are from 65% to 88% and 98% to 100%*, respectively^10,13^*. The color temperature of CFLs and incandescent lamps are 2900-6500 °K and 2500-3000° K, respectively is reported*^10^*. The color temperature of incandescent lamps were from 2569 to 2760 °K*^11^*. The color temperature of CFLs are from 2700 to 6500 °K and the color temperature of inflamed lamps are near to 2800 °K*^9,12^. Researchers introduced three bands for low consumption cool white lamps including 435 nm, 550 nm and 610 nm^12, 13^.

CFLs usually generate ultraviolet (UV) emissions from a discharge in mercury vapor. However, the phosphor coating inside the glass envelope of the lamp converts the energy of the ultraviolet photons into visible radiation. However, some ultraviolet emissions are transmitted through defects in the phosphor coating and the glass envelope,^14^. By changing the composition and thickness of the phosphor and the glass envelope, a wide variety of ultraviolet emission spectrums can be created. On the other hand, the UV emissions of incandescent lamps are influenced by the filament temperature and the bulb absorption^15^. Under normal use, the UV emission at distance 65 cm from CFLs would not produce a significant UV hazard^16^. Both daylight and cool white fluorescent lamps could emit UVA and UVB radiations along with low health risks^17^. The UVC emission was considerably lower than UVB and UVA emissions,^18^. Typical CFLs frequently emit amount of UVB (280-315 nm), UVA (315-400 nm) and infrared (> 760 nm) radiations^18^.

Reducing energy consumption is main factor for broad use of the compact fluorescent lamps by Iranian user in homes, industries, malls, hospitals, universities, offices and etc. This study aimed to investigate the light emissions of some CFLs commercially available to the Iranian consumers. The characteristics of existing incandescent lamps were also investigated and their results were compared with those of CFLs.

## Methods

In this cross sectional study, 60 lamps including 48 single envelope compact fluorescent lamps, and 12 incandescent lamps commercially available to the Iranian consumers were randomly selected from different manufacturers. The compact fluorescent lamps were included warm white and daylight marked from 11 to 40 W. Lighting characteristics including color rendering index, correlated color temperature and spectral power distribution were measured using spectroradiometer (model Light Spex-3‏ -Gretag Macbeth) in the Institute for Color Science and Technology (Tehran- Iran) between 2014 and 2015.

**Figure 1:**
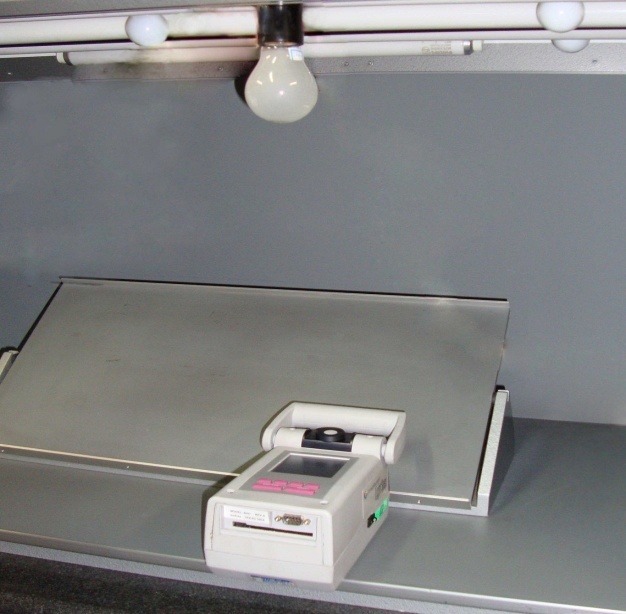
The experiment setup for measurement of lighting source characteristics.

The spectroradiometric measurement of light sources was perfomed based on CIE 63-1984 method recommended by International Electrotechnical Commission (IEC)^19^. The employed spectroradiometer is periodically calibrated using a standard source of irradiance based on standard method ^20^. Spectroradiometers are the most accurate for measuring spectral energy distribution of any light source. They are used for determining not only the radiometric and photometric quantities, but also the colorimetric quantities of a lighting source. The setup of lighting quality experiments for studied lamps is shown in Figure 1. In this way, the studied lamps were separately installed at a distance of half a meter above the spectroradiometer in a grey box and then after five minutes, the interested data for each lamp was registered.

UV emissions of the studied lamps were also measured with HAGNER digital radiometer, model EC1-X. Measurements of UV emission were conducted at minimum distance for use 25 cm and maximum distance for use 200 cm using a calibrated radiometer in the dark room with the conditioned surfaces according to the International Commission on Non-Ionizing Radiation Protection (ICNIRP) recommendations ^15^.

The ICNIRP guideline for maximum human biologically efficient radiant exposure of the eye and skin to actinic UV within an 8 h period is 30 J m^2^ effective. If the irradiance is constant, the permissible exposure duration, t_max_ (s) is the ICNIRP exposure limit of 30 J m^2^ divided by the effective irradiance (E) in W m^2^ as shown in equation 1.

(1) $$t_{\max}=\frac{30\;j\;m^{2}}{E_{effec}(w\;m^{2})}$$

Following the ICNIRP guidelines, the ocular exposure is also limited to unweighted UVA, 10 kJ m^2^, for periods up 8 h workday. If the irradiance is constant, the permissible exposure duration, t_max_ (s) is the ICNIRP exposure limit of 10 kJ m^2^ divided by the effective irradiance (W m^2^) (Equation 2).

(2) $$t_{\max}=\frac{10\;kj\;m^{2}}{E_{effec}(w\;m^{2})}$$

Occupational exposure limit of UVA and UVB for periods up 8 h are 0.33 W m^2^and 0.001 W m^2^following the ICNIRP guideline.Moreover, public exposure limit of UVA and UVB for periods up 16 h are 0.174 W m^2^and 0.0005 W m^2^, respectively ^15^.

CIE S009 (CIE 2006a) was developed a manufacturer’s standard to specify risk groups, to be assigned to the lighting lamp by the manufacturer. The risk group definitions are based on changing the maximum permissible exposure durations^21^. IEC defined four groups based on UVB exposure at distance 25 cm including no risk group; permissible exposure duration up to 30000 s, risk group 1; permissible exposure duration up to 10000 s, risk group 2; permissible exposure duration up to 1000 s, risk group 3; permissible exposure duration lower than 1000 s ^21^. Data analysis was performed using SPSS16 software (Chicago, IL, USA).

## Results

Lighting characteristics of the CFLs and incandescent lamps including correlated color temperature and color rendering index are shown in Table 1. The differences of CRI values based on the CFLs types were not statistically significant (*P*=0.19). However, the differences of CCT values based on the CFLs types were statistically significant (*P*=0.001). Moreover, color rendering indices of incandescent lamps were more than the CFLs. Daylight (cool white) CFLs had more desirable color temperature than the others. Sunlight had color temperature approximately between 5800 – 6500 (°K) in clear sky.

**Table 1:** The lighting characteristics of the compact fluorescent lamps and incandescent lamps.

**Lighting characteristics**	**Warm white lamps**		**Daylight lamps**		**Incandescent lamps**		***P*** ** value**
	**Mean**	**SD**	**Mean**	**SD**	**Mean**	**SD**	
Color rendering index (%)	81.80	0.67	80.10	3.70	99.60	0.09	0.190
Correlated color temperature(°K)	2730	64.30	6388	10.83	2748	12.80	0.001

**Figure 2:**
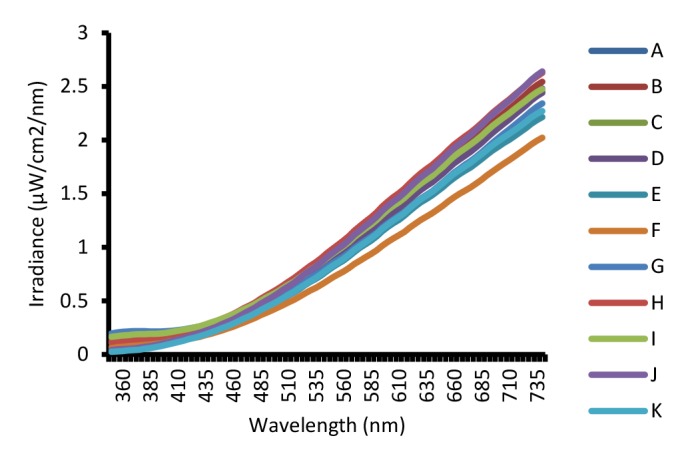
Spectral power distribution of warm white CFLs

**Figure 3:**
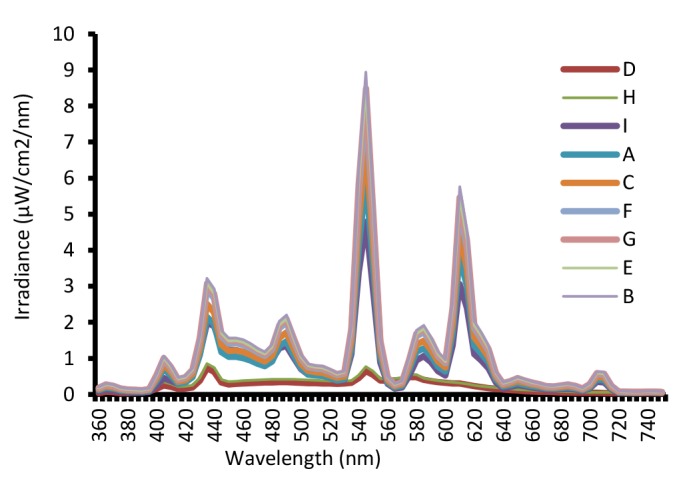
Spectral power distribution of daylight (cool white) CFLs

**Figure 4:**
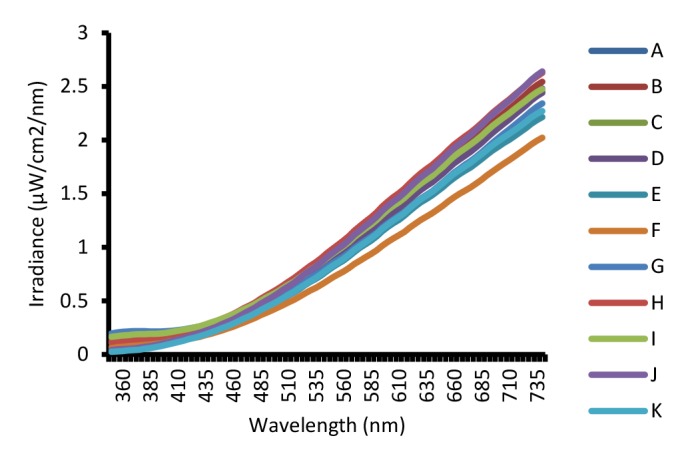
The spectral power distribution of incandescent lamps.

Figures 2, 3 and 4 show the spectral power distributions of warm white, daylight (cool white) CFLs and incandescent lamps. SPD showed the graphic representation of the radiant power emitted by the different lighting lamps (A, B, C, …) at each wavelength over the visible region. This curve provides the user with a visual profile of the color characteristics of a light source. The SPD diagrams for the CFLs showed typical fluorescent response, with a few narrow spectral peaks included 435, 545 and 610 nm rising above the overall curve.

Moreover, the incandescent lamps showed high power in the longer wavelengths of the visible spectrum (Figure 4). UV emissions of the CFLs and incandescent lamps are shown in Table 2. National exposure limit for exposure to UV radiation was according to the ICNIRP guidelines. The occupational and public exposures to UVA emissions from the studied CFLs and incandescent lamps at any distances were lower than the ICNIRP exposure limits. However, occupational exposures for periods up 8 h to UVB emissions from the studied CFLs and incandescent lamps as local lighting sources at distances up to 0.25 m were more than the ICNIRP exposure limits. Moreover, public exposures for periods up 16 h to UVB from the studied CFLs and incandescent lamps at any distances up to 2 m were not safe compared with the ICNIRP exposure limits.

**Table 2:** Ultraviolet emissions of the compact fluorescent lamps and incandescent lamps at the defined distances.

**Type of lamps**	**UVA(W/m** ^ 2 ^ **)**				**UVB(W/m** ^ 2 ^ **)**			
	**Mean**	**SD**	**Min**	**Max**	**Mean**	**SD**	**Min**	**Max**
**Compact fluorescent lamps**								
25 cm	0.0356	0.0205	0.0081	0.0857	0.0042	0.0051	0.0003	0.0268
200 cm	0.0013	0.0009	0.0002	0.0033	0.0005	0.0003	0.0001	0.0011
**Incandescent lamps**								
25cm	0.0170	0.0030	0.0100	0.0200	0.0024	0.0004	0.0001	0.0040
200 cm	0.0010	0.0001	0.0001	0.0010	0.0005	0.0001	0.0001	0.0010

The calculated exposure duration, t_max_ (s) for the studied CFLs based on equation 1 following the ICNIRP exposure limit was equal to 7142 s. Therefore, the studied CFLs can be classified as risk group 1 (along with permissible exposure duration up to 10000 s).

## Discussion

Lighting comfort is a main factor affecting our ability to perform tasks in closed spaces. Some characteristics of lighting sources can effect on quality of lighting. The development of CFLs and the eventual replacement of incandescent lamps have benefits for the public, but some concerns are existed about their light characteristics and health risks.

The current study showed the color rendering indexes of the CFLs were approximately 80.8% ± 2.9% relatively acceptable for general tasks in viewpoints of color properties. As expected, the incandescent lamps have the color rendering indices near to maximum value CRI of 100. The lower CRI values indicate that some colors may appear unnatural when light is emitted by the lamp. Based on color rendering, a lamp with a CRI of 90 is desirable and can be used for tasks requiring the most accurate color discrimination ^7^.

Based on the results of CCT values, the daylight CFLs have more natural color temperature than the other types of the studied lamps. Warm white light (CCT near 3000^0^ k) facilitates relaxation and improves human’s well-being and creates nice atmosphere, while daylight or cool white light (CCT near 5000^0^ k) stimulates and activates the human body^23^. Therefore, warm white lamp can be employed for homes usage, while daylight lamp can be used for offices rooms.

In each lamp type, the values of CRI and CCT showed some differences. This means that the qualities of lamps produced by manufacturers are not the same. The most important factors determining the light quality of CFLs are the type and combination of the used phosphors. Some phosphors produce white light when employed alone. However, a combination of phosphors with different characteristics frequently is used. In regard to a broad range of phosphors available, CFLs are produced in a wide range of colors characteristics.

Spectral power distribution of daylight (cool white) and warm white CFLs showed a few spikes on same certain wavelengths. These spikes indicate some parts of the color spectrum have main role in the color rendering for objects illuminated through lighting sources. Duff examined the use of compact fluorescent lamps for the domestic environment and showed the CFLs have three peaks included 440, 540 and 620 nm in spectral power distribution which were approximately similar to our results^24^.

The incandescent lamps have high power in the longer wavelengths of the visible spectrum. Generally, continuous spectrum or very full-line spectra produce less distortion of object colors than a few discrete lines. On the other hand, sunlight as a cool light source with a high color temperature usually emits large amounts of energy in the blue and green part of the spectrum. Therefore, incandescent lamps with smooth SPD curves are not like sunlight and do not render blues, adequately.

Currently, due to a little data related to UV emission of CFLs, public concerns about the potential of UV exposure from compact fluorescent lamps have been increased. Moreover, patients with photosensitive dermatologic and systemic diseases often question regarding the ultraviolet emissions of residential lighting sources. Ultraviolet radiation can be hazardous to patients with photosensitive skin disorders, such as lupus erythematosus, xeroderma pigmentosum and skin cancer^25^. ICNIRP exposure limits are protective for normal human skin types; however they cannot be applied to people with highly photosensitive skin. Therefore, the other objectives of the current study were to compare the levels of ultraviolet emissions among lighting sources.

Occupational exposures for periods up 8 h to UVB emissions from the studied lamps at distances up to 0.25 m were more than the ICNIRP exposure limits. Consequently, UV emissions from the studied CFLs with single envelopes can result in overexposure of the skin when the lamps are used as local lighting on work tables. The European commission scientific committee on emerging and newly identified health risks (SCENIHR) stated that UV emitted by single envelope CFLs at distances of less than 25 cm can lead to ultraviolet exposures near to the current exposure limits set to protect peoples from skin and retinal damage^26^.

Our results showed that increase the distance from the CFLs, results in to reduce the level of UV exposure. UVB and UVA emissions at 100 and 150 cm from CFLs are negligible^22^. People can utilize double envelope or encapsulated CFLs for desktop use at distances closer than 25 cm. On the other hand, continuous public exposures for periods up 16 h to UVB from the studied CFLs and incandescent lamps at any distances up to 2 m are more than the ICNIRP exposure limits. Therefore, for residential usage, public exposure limits must be considered for determining the ideal installation height of lamps. Although, the incandescent lamps emit measureable amounts of UVA, the UVB, but these emissions were lower than the UV emissions of the CFLs. The amount of UVB emitted from single envelope CFLs, from the same distance of 20 cm, was nearly ten times higher than that incandescent lamp^27^. The studied CFLs classified as risk group 1 presented a low level of risk to the normal skin but are potentially harmful to photosensitive patients. The ultraviolet radiation emitted from the double envelope CFLs were much lower compared with the single envelope CFLs^28,29^. The second envelope can block most of the UV emitted from the lamps. Double envelope CFLs are therefore a safer alternative for UV sensitive peoples. It should be noted that, a pilot study showed UVC emissions of CFLs and incandescent lamps are not considerable like the results of other studies related to UVC emissions of lighting sources ^17, 18, 22^. In current study, UVC emissions were not observed at the distance 25 cm from lamps.

It is also suggested that light emitting diodes as an alternative local lighting option that emit little or no UV radiation for people with photosensitive skin disorders. The cover around CFLs can also protect people from the possibility of direct UV exposure in all possible positions. Covers materials such as plastics are more absorbent of UV than glass. Moreover, for using CFLs as local lighting, moving desk lamp slightly further away is a very effective method of reducing UV exposure. There is very little information on blue light of CFLs which may cause a photochemical injury to the retina, called blue-light hazard. However, the blue light hazard of CFLs on normal skin has not been completely investigated ^30^ and further researches are required from health effects viewpoints.

## Conclusions

The widespread use of compact fluorescent lamps is followed by people concerns about their light qualities and health risks. Daylight CFLs have more desirable and natural color temperature compared with the other types of the studied lamps. Therefore, warm white lamps can be employed for homes usage, while daylight lamps can be used for offices rooms. Our results confirmed using single envelope CFLs for long periods of time near the body at distances of less than 25 cm can result in overexposure to actinic ultraviolet emissions. The use of double envelope lamps can efficiently reduce UV emissions and the risk of health hazards especially for sensitive people. Generally, the single envelope CFLs must be used at distances greater than 25 cm for occupational exposure and upper than 200 cm for public exposure. Moreover, public education on selection of lighting lamps for specific uses is essential for the residential, commercial and industrial applications. The national regulatory agencies should also prevent the informal productions of lighting sources and support manufactures for improvement of production technology.

## Acknowledgments

This study was financially supported by Vice President for Research in Hamadan University of Medical Sciences (project number: 92021493). The authors announce their greetings from Mrs. Khalili, the director of Color Physics Lab in the Institute for Color Science and Technology (Tehran-Iran).

## Conflict of interest statement

The authors have no conflict of interests to declare

## Highlights

- The widespread use of compact fluorescent lamps is followed by people concerns about their health risks.
- Public education on selection of lighting sources is essential for the residential applications.
- Warm white lamps can be employed for homes usage, while daylight lamps can be used for offices rooms.
- CFLs must be used at distances greater than 200 cm for public exposure.

## References

[1] Davis W. Measuring color quality of light sources. 6th International Conference on Solid State Lighting; September 12; California 2006.

[2] Hoffmanna G, Guflera V, Griesmacherb A, Bartenbach C, Canazeic M, Stagglc S (2008). Effects of variable lighting intensities and colour temperatures on sulphatoxymelatonin and subjective mood in an experimental office workplace. Appl Ergon.

[3] Ohno Y.Optical metrology for LED and solid state lighting. Fifth Symposium Optics in Industry;September 8-9; Santiago de Queretaro; 2006.

[4] Pal Singh L, Katal G (2013). A comparative study on design and operation of fluorescent lamps, cfls and leds. IntJ Eng Res Appl.

[5] Luo MR (2011). The quality of light sources. Color Technol.

[6] Beke L, Kranicz B, Schanda J.Simulating colour appearance under different illuminants–Possible use to describe light source colour quality. In Proceedings of AIC Meeting; June 15- June 18; Stockholm 2008.

[7] Illuminating Engineering Society of North America (IESNA). Lighting handbook. New York: IESNA; 2000.

[8] Smith BA. Lighting for health and safety. Massachusetts: Butterworth-Heinemann; 2000.

[9] Gloria SC, Sproul AB, Dain SJ. Performance of ‘energy efficient’ compact fluorescent lamps. Clin Exp Optom2010; 93(2):66-76. 10.1111/j.1444-0938.2010.00462.x 20132232

[10] Majoros A. Artificial lighting. Budapest: Budapest University of Technology and Economics; 2011.

[11] Yuen GSC, Sproul AB, Dain SJ (2010). Performance of energy efficientcompact fluorescent lamps. Clin Exp Optom.

[12] Sandor N, Schanda J (2006). Visual colour rendering based on colour difference evaluations. Lighting Res Technol.

[13] Khazova M, OHagan JB (2008). Optical radiation emissions from compact fluorescent lamps. Radiat Prot Dosim.

[14] Mironava T, Hadjiargyrou M, Simon M, Rafailovich MH (2012). The effects of UV emission from compact fluorescent light exposure on human dermal fibroblasts and keratinocytes in vitro. Photochem Photobiol Sci.

[15] International Commission on Non Ionizing Radiation Protection (ICNIRP) (2004). Guidelines on limits of exposure to ultraviolet radiation of wavelength between 180 nm and 400 nm. Health Phys.

[16] Whillock M, McKinlay A, Kemmlert J, Forsgren P (1990). Ultraviolet radiation emission from miniature compact fluorescent lamps. Lighting Res Technol.

[17] Sayre R, Dowdy J, Poh-Fitzpatrick M (2004). Dermatological risk of indoor ultraviolet exposure from contemporary lighting sources. Photochem Photobiol Sci.

[18] Fenton L, Ferguson J, Moseley H (2012). Analysis of energy saving lamps for use by photosensitive individuals. Photochem Photobiol Sci.

[19] International Electrotechnical Commission (IEC). Spectroradiometric measurement of light sources. Vienna: CIE Publication;1984.

[20] ASTM G138. Standard test method for calibration of a spectroradiometer using a standard source of irradiance. Pennsylvania: West Conshohocken; 2012.

[21] International Electrotechnical Commission (IEC). Photobiological safety of lamps and lamp systems. Geneva: IEC; 2006.

[22] NuzumKeim AD, Sontheimer RD (2009). Ultraviolet light output of compact fluorescent lamps: comparison to conventional incandescent and halogen residential lighting sources. Lupus.

[23] Yasukouchi A, Ishibashi K (2005). Non-visual effects of the color temperature of fluorescent lamps on physiological aspects in humans. J Physiol Anthropol Appl Human Sci.

[24] Duff JT (2012). An examination in to the use of compact fluorescent lamps in the domestic environment. J Sust Eng Design.

[25] Klein RS, Werth VP, Dowdy JC, Sayre RM (2009). Analysis of compact fluorescent lights for use by patients with photosensitive conditions. Photochem Photobiol Sci.

[26] Scientific Committee on emerging and newly identified health risks (scenihr). Health effects of artificial light. Belgium: European Commission; 2012.

[27] Eadie E, Ferguson J, Moseley H (2009). A preliminary investigation into the effect of exposure of photosensitive individuals to light from compact fluorescent lamps. Br J Dermatol.

[28] Shama P, Jaiswal VK, Kandpal HC (2009). Ultraviolet radiation emitted by compact fluorescent lamps. J Metrol Soc.

[29] Fenton L, Moseley H (2014). UV emissions from low energy artificial light sources. Photodermatol Photoimmunol Photomed.

[30] Okuno T, Saito H, Ojima J (2002). Evaluation of blue-light hazards from various light sources. Dev Ophthalmol.

